# Clinical outcomes in elderly patients on direct oral anticoagulant drugs undergoing surgery for acute hip fracture (AHF)

**DOI:** 10.1007/s00068-025-03016-0

**Published:** 2025-11-25

**Authors:** Elsa Wemmert, Pernilla Eklöf, Victor Linder, Emilia Möller Rydberg, Keti Dalla, Bengt Nellgård, Fredrik Hessulf, Adam Piasecki

**Affiliations:** 1 Institute of Clinical Sciences, Department of Anaesthesiology and Intensive Care, Sahlgrenska Academy, University of Gothenburg, Gothenburg, Sweden; 2 Department of Anaesthesiology and Intensive Care, Sahlgrenska University Hospital, Göteborgsvägen 31, Mölndal, 43130 Sweden; 3 Department of Internal Medicine, Geriatrics and Emergency Medicine, Sahlgrenska University Hospital, Göteborgsvägen 31, Mölndal, 43130 Sweden; 4 Institute of Clinical Sciences, Department of Orthopaedics, Sahlgrenska Academy, University of Gothenburg, Gothenburg, Sweden; 5 Department of Orthopaedics, Sahlgrenska University Hospital, Göteborgsvägen 31, Mölndal, 43130 Sweden

**Keywords:** Acute hip fracture, Mortality, Geriatric patients, Anticoagulants, Direct oral anticoagulants, DOAC

## Abstract

**Methods:**

This single-center retrospective observational study analyzed 594 elderly acute hip fracture (AHF) patients surgically treated in 2023. The primary aim was to determine if direct oral anticoagulant (DOAC) therapy in elderly AHF patients is associated with higher mortality 360 days after surgery. Secondary aims were to assess whether DOAC-users have a longer time to surgery and/or higher transfusion requirements than non-users. Numerical outcomes were analyzed using the Mann-Whitney U test, and categorical outcomes using the Chi-squared test. To analyze mortality, Kaplan-Meier survival curves and multivariate Cox regression were used.

**Results:**

170 AHF patients (28.6%) were treated with DOACs. DOAC-users had a higher mortality 360 days after surgery compared to non-users (35.9% vs. 16.7%, *p* < 0.001). This association remained significant after adjusting for covariates (HR = 1.57, *p* = 0.02). Further, patients using DOACs had a longer time to start of surgery (24.6 h vs. 22.6 h, *p* = 0.0048) and higher transfusion rates (51.2% vs. 39.4%, *p* = 0.009), while transfusion volumes did not differ (*p* = 0.091).

**Conclusion:**

Surgically treated acute hip fracture patients on DOACs had higher 360-day mortality, adjusted for age, sex, comorbidity and frailty. Additionally, these patients experienced longer time to surgery and higher transfusion rates. This study provides important insights into the perioperative outcomes of elderly AHF patients using DOACs and highlights the need for further research.

## Key summary points

Aim: This study aims to examine whether direct oral anticoagulant (DOAC) therapy, in elderly acute hip fracture (AHF) patients, is associated with higher mortality 360 days after surgery.

Findings: DOAC-users had a significantly higher 360-day mortality. DOAC use was identified as an independent predictor of mortality.

Message: The findings highlight the challenges of managing AHF patients on DOAC therapy and inspire future research to improve outcome for this vulnerable group of patients.

## Introduction

Acute hip fractures (AHF) are common in the elderly and constitute a major public health challenge [1]. The consequences of AHF are severe including impaired quality of life, functional decline and substantial loss of independence [2, 3]. Furthermore, patients who sustain an AHF have high one-year mortality rates ranging between 18% and 31%, with higher rates observed in men [4].

AHF can be divided into three major groups based on the specific location: femoral neck, per-/intertrochanteric and subtrochanteric [5]. Surgery is the standard treatment for AHF, aiming at alleviating pain, restoring function, and minimizing complications associated with prolonged immobility [6]. Early operative intervention (within 24–48 h) has been shown to improve return to independence and lowering the risk of death and postoperative complications [7–9]. Contrary, delaying surgery for > 48 h has been associated with significantly increased mortality as well as complications such as pressure sores [9, 10]. Thus, the current guidelines generally suggest that AHF surgery should be performed within 24–48 h [11, 12].

Anticoagulant use is common in AHF patients due to their cardiovascular comorbidities (e.g. atrial fibrillation) with associated risk for thromboembolism [13]. In recent years, there has been a shift from traditionally used vitamin K antagonists (VKAs) to direct oral anticoagulants (DOACs) [14, 15], including in acute hip fracture patients [16]. DOACs, as compared to VKAs, offer a more favorable pharmacological profile [14, 17], are more effective in stroke prevention and have fewer complications [18]. The reported prevalence of DOAC usage among AHF patients varies between 1% and 20% [10].

However, in an acute surgical setting, the use of DOACs presents a challenge in balancing the risk of excessive bleeding with the benefits of early surgical intervention. This is partly due to a half-life dependent on renal function, the absence of any standardized monitoring test for DOACs (in contrast to the International Normalized Ratio used for VKAs), and limited availability of reversal agents. Previous studies have shown that AHF patients on DOACs experience increased time to surgery [10, 19]. Swedish and European guidelines recommend discontinuing DOACs 24–48 h prior to surgical intervention or neuraxial anesthesia [20, 21]. In case of renal impairment or high dose DOAC, discontinuation up to 96 h is sometimes recommended. The extended waiting time can make surgery within 24–48 h with neuraxial anesthesia impossible. AHF are sudden and unplanned events, where patients typically present without prior discontinuation of anticoagulant therapy, which adds complexity to perioperative management.

While several studies investigating perioperative outcomes in AHF patients have shown increased mortality [22–24] and transfusion rates [19, 25] in DOAC-treated patients, other studies have reported conflicting results [25]. However, many of these studies have been limited by small sample sizes or lack of adjustment for confounding variables. In this study, we aimed to evaluate clinical outcomes: mortality 360 days after surgery, time to surgery and transfusion requirements, in elderly patients on DOACs undergoing surgery for an isolated AHF. Also, by adjusting for key confounders in the mortality analysis, our study aims to contribute to a better understanding of outcomes in this patient group.

## Methods

### Study design

This is a retrospective observational study using data collected from the electronic health records (EHR) of the Sahlgrenska University Hospital Mölndal in Sweden. The EHR search criteria and all variables collected from EHR are presented in Appendix Table 1.

## Inclusion and exclusion criteria

**Fig. 1:**
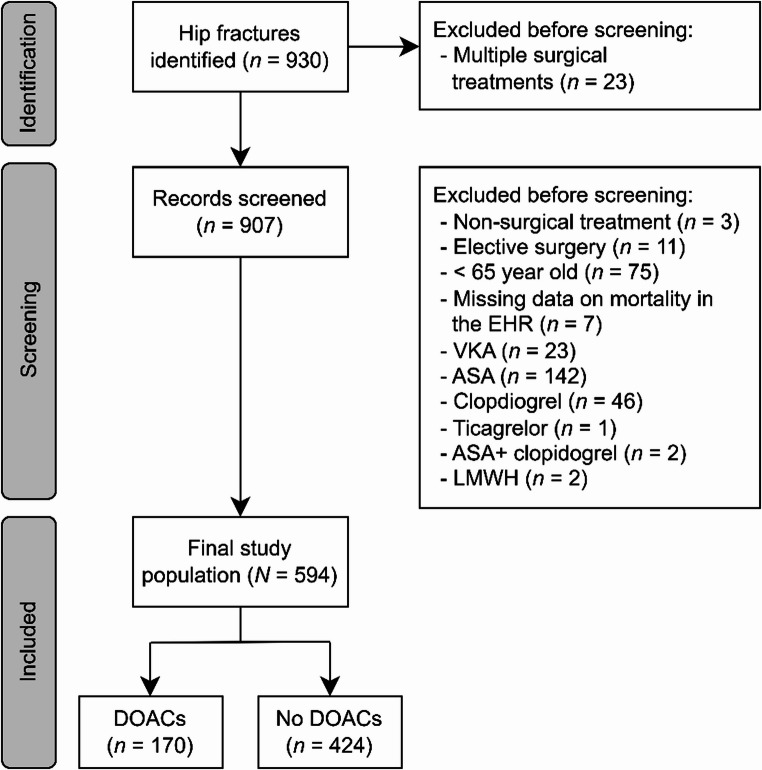
Flowchart of the study population formation

A detailed overview of the study population formation process is presented in Fig. 1. Patients ≥ 65 years of age with a Swedish social security number undergoing acute surgery for an isolated hip fracture between 1 January 2023 and 31 December 2023 were included. The exclusion criteria were age < 65 years, preoperative use of other anticoagulation or antiplatelet therapies, elective or no surgical treatment, and missing data on mortality in the EHR. Patients receiving other forms of anticoagulation or antiplatelet therapy preoperatively were excluded due to differing mechanisms of action, the predominance of DOAC use in our population, and the aim to maintain a homogeneous study cohort. In cases where a patient underwent multiple AHF surgeries during the study period, only data from the first surgery was included. Given the hospital’s profile, it does not admit or treat acute multitrauma patients.

*EHR* Electronic health records, *VKA* Vitamin K antagonist, ASA Acetylsalicylic acid, LMWH Low molecular w weight heparin, DOACs Direct oral anticoagulants.

## Outcomes

The primary outcome for all study groups was mortality 360 days after surgery; secondary outcomes were transfusion rate, transfusion volume and time to surgery. The transfusion rate was presented as the percentage of patients who received at least one red blood cell transfusion from admission to discharge. Transfusion volume was reported as the total volume of red blood cells transfused, in milliliters, during the same period. Time to surgery was defined as the time from the first diagnostic imaging confirming the diagnosis to surgery.

### Statistical analysis

For the statistical analyses we used: JASP (JASP Team) version 0.19.1, R version 4.4.1 and RStudio 2024.12.1 Build 563. Data were presented as absolute frequencies, percentages and medians with interquartile range (IQR). Normality was assessed using Shapiro-Wilk test. Non-parametric tests were used because normality assumptions were not satisfied for most of the variables.

For comparisons between the study groups, we used the Mann Whitney U test (for continuous variables) or the Chi-squared test (for categorical variables). A p value ≤ 0.05 was considered statistically significant.

To detect differences in 360-day mortality between the groups, the Kaplan-Meier survival curves were built and compared with a two-sided log-rank test.

A multivariate Cox proportional hazards regression analysis was conducted to investigate the association between DOAC treatment and 360-day mortality, while adjusting for relevant covariates. The number of covariates was set at five, based on the number of events (deaths) [26]. The proportional hazards assumption was assessed using Schoenfeld residuals. This revealed two significant violations for gender and Clinical Frailty Scale (CFS) which were addressed by stratifying the model by these variables. The independent variables included in the model were age, gender, DOAC usage, CFS (categorized as 1–4 vs. 5–9) and ASA score (categorized as 1–2 vs. 3–4). These variables were selected based on clinical expertise and earlier studies on AHF patients using regression models [27, 28]. We checked for multicollinearity using variance inflation factor and tolerance. Patients with missing data in one or more of the variables were excluded from the analysis. The Cox regression model provided adjusted hazard ratios (HR) with 95% confidence intervals (CI). The *p* values for each independent variable were calculated with the Wald test, and the overall significance of the model was determined with a likelihood ratio test.

## Results

### Baseline characteristics

A total of 594 AHF patients were included in the study, of whom 170 (28.6%) were treated with DOACs, and 424 (71.4%) were not. Patient characteristics and preoperative laboratory values are presented in Table 1.

**Table 1:** Patient characteristics and preoperative laboratory values

	DOACs	No DOACs	*p* value^1^
**Total patients (*N*** **= 594)**, ***n***	170	424	
**Age**,** median (IQR)**	87 (82–91)	82 (76–89)	< 0.001
**Gender**, ***n*** **(%)**			0.023
Male	63 (37.1%)	117 (27.6%)	
Female	107 (62.9%)	307 (72.4%)	
**BMI**,** median (IQR)**	23.7 (21.7–26.2)	22.6 (20.1–25.5)	0.002
**ASA score**, ***n*** **(%)**			< 0.001
1–2	23 (13.5%)	226 (53.3%)	
3–4	146 (85.9%)	198 (46.7%)	
Missing data	1 (0.6%)	0 (0%)	
**CFS**, ***n*** **(%)**			0.001
1–4	34 (20%)	145 (34.2%)	
5–9	110 (64.7%)	232 (54.7%)	
Missing data	26 (15.3%)	47 (11.1%)	
**Type of DOAC**, ***n*** **(%)**			
Apixaban	143 (84.1%)		
Rivaroxaban	14 (8.2%)		
Dabigatran	10 (5.9%)		
Edoxaban	3 (1.8%)		
**Type of hip fracture**, ***n*** **(%)**			0.382
Femoral neck	89 (52.3%)	243 (57.3%)	
Intertrochanteric	72 (42.4%)	154 (36.3%)	
Subtrochanteric	9 (5.3%)	27 (6.4%)	
**Type of surgical treatment**			< 0.001
Hemiarthroplasty	69 (40.6%)	138 (32.5%)	
Total arthroplasty	7 (4.1%)	50 (11.8%)	
Intramedullary nail	74 (43.5%)	69 (16.3%)	
Other internal fixation methods	20 (11.8%)	167 (39.4%)	
**Type of anesthesia**, ***n*** **(%)**			< 0.001
Spinal	144 (84.7%)	399 (94.1%)	
General	21 (12.4%)	18 (4.2%)	
Combined (neuraxial and general)	5 (2.9%)	6 (1.4%)	
Missing data	0	1 (0.2%)	
**Hemoglobin (g/L)**,** median (IQR)**	121.5 (111–132)	124 (116–135)	0.022
**Creatinine (µmol/L)**,** median (IQR)**	83.5 (65–112)	70 (58–86)	< 0.001
**eGFR (mL/min/1**,**73 m2)**,** median (IQR)**	50 (37–64)	63 (50–73)	< 0.001
**NT-proBNP (ng/L)**,** median (IQR)**	1660 (695–3660)	324.5 (183–859.3.3)	< 0.001

DOACs Direct oral anticoagulants, IQR Interquartile range, BMI Body mass index, ASA score American Society of Anesthesiologists, eGFR Estimated glomerular filtration rate score, Clinical Frailty Scale

^1^p values were calculated using the Mann Whitney U test (for continuous variables) and the Chi-squared test (for categorical variables)

The DOAC group was significantly older (median: 87 years [IQR: 82–91] vs. 82 years [IQR: 76–89], *p* < 0.001), had a higher proportion of males (37.1% vs. 27.6%, *p* = 0.023), and a higher body mass index (BMI) (median: 23.7 [IQR: 21.7–26.2] vs. 22.6 [IQR: 20.1–25.5], *p* = 0.002) compared to the non-DOAC group.

Furthermore, patients on DOAC therapy had greater comorbidity and frailty, as indicated by significantly higher ASA scores (85.9% ASA 3–4 vs. 46.7% in the non-DOAC group, *p* < 0.001) and CFS (64.7% CFS 5–9 vs. 54.7%, *p* = 0.001).

Apixaban was the most frequently used DOAC (84.1%), followed by Rivaroxaban (8.2%), Dabigatran (5.9%) and Edoxaban (1.8%). Femoral neck fractures were the most common type of fractures in both groups (52.3% in the DOAC group vs. 57.3% in the non-DOAC group), followed by intertrochanteric (42.4% vs. 36.3%) and subtrochanteric fractures (5.3% vs. 6.4%). However, the difference in hip fracture types between the groups was not significant (*p* = 0.382). The type of surgical treatment differed significantly between groups (*p* < 0.001), with intramedullary nails being the most used in the DOAC group (43.5% vs. 16.3%), while other internal fixation methods were most frequently used in the non-DOAC group (39.4% vs. 11.8%). The most common form of anesthesia in both groups was spinal anesthesia (84.7% vs. 94.1%), whereas general and combined anesthesia were used less frequently (12.4% vs. 4.2% and 2.9% vs. 1.4%). The type of anesthesia differed significantly between the groups (*p* < 0.001).

Preoperative lab values showed significant differences between patients in the DOAC and those in non-DOAC group. The patients using DOACs had lower hemoglobin (median: 121.5 [IQR: 111–132] vs. 124 [116–135], *p* = 0.022), higher creatinine (median: 83.5 [IQR: 65–112] vs. 70 [58–86], *p* < 0.001), lower eGFR (median: 50 [IQR: 37–64] vs. 63 [IQR: 50–73], *p* < 0.001) and higher NT-proBNP levels (median: 1660 [IQR: 695–3660] vs. 324 [IQR: 183–859.3.

### Mortality 360 days after surgery

**Fig. 2:**
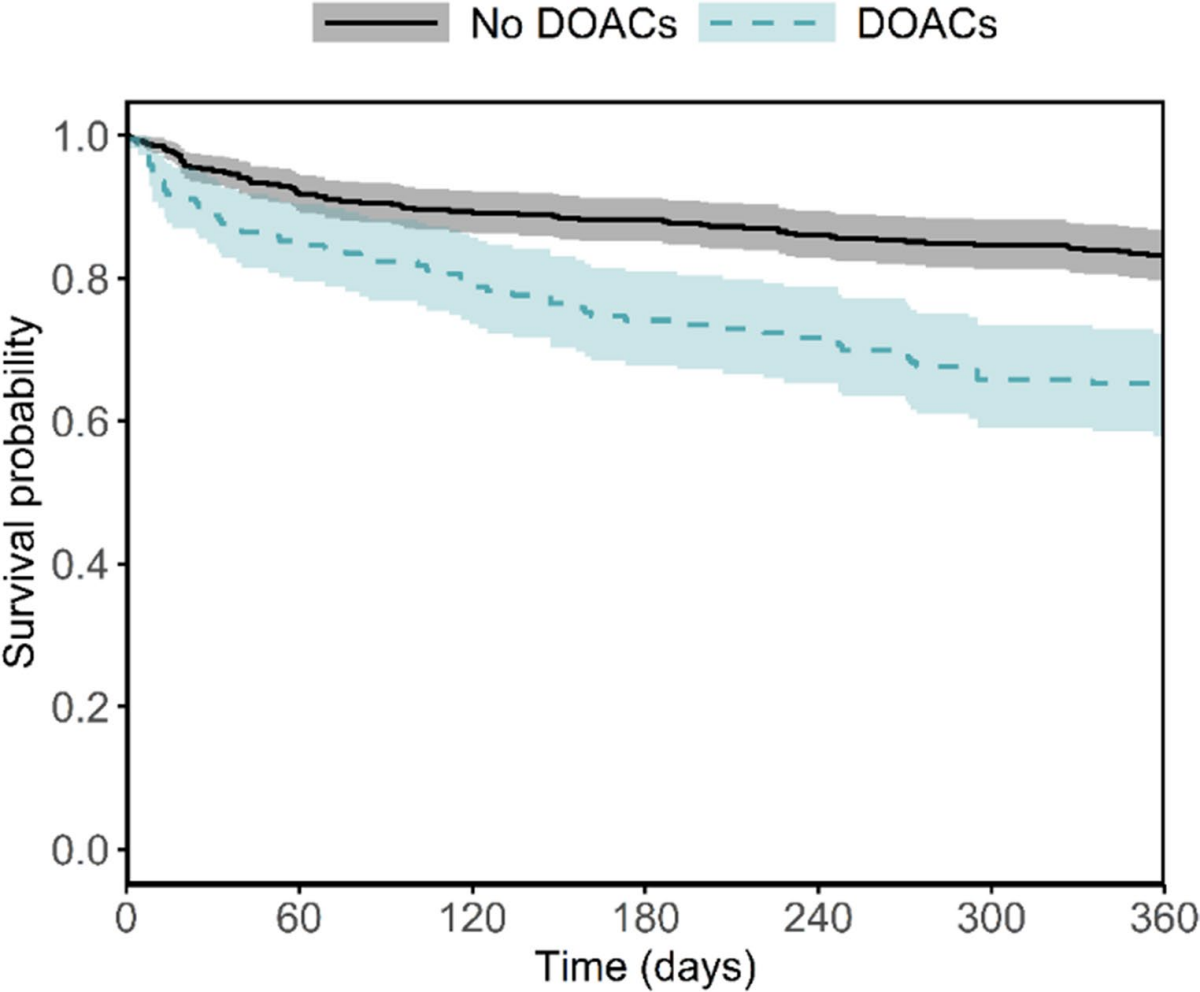
360-day mortality. At 360 days, the survival rate was 64.1% for patients using DOAC and 83.3% for the patients not using DOACs. The survival differed significantly between the groups (*p* < 0.001, log-rank test)

The Kaplan-Meier curves demonstrate that the survival started to differ early, remaining consistently lower in the DOAC group of patients throughout the 360-day follow-up (Fig. 2). During this period, 132 deaths were recorded in total, with 61 deaths occurring in the DOAC group and 71 deaths in the non-DOAC group. At 360 days, the survival rate was 64.1% (95% CI: 57.3–71.7) for patients using DOAC and 83.3% (95% CI: 79.8–86.9) for the patients not using DOACs. Consequently, the mortality rate was 35.9% vs. 16.7%. The survival in the two groups differed significantly (*p* < 0.0001, log-rank test).

#### *DOACs* direct oral anticoagulants

The use of DOACs was significantly associated with an increased risk of mortality at 360 days (HR = 1.57, 95% CI: 1.07–2.29, *p* = 0.02) (Table 2). A higher ASA score (HR = 1.87, 95% CI: 1.10–3.16, *p* = 0.02), and age (HR = 1.05, 95% CI: 1.02–1.08, *p* < 0.001) all showed a significant association with increased mortality.

**Table 2:** Predictors of 360-day mortality calculated using a Cox proportional hazards model (stratified by gender and clinical frailty scale)

	*n*	HR	95% CI	*p* value
No DOAC	377			Reference
DOAC	143	1.57	1.07–2.29	0.02
Age	520	1.05	1.02–1.08	< 0.001
ASA score 1–2	221			Reference
ASA score 3–4	299	1.87	1.10–3.16	0.02

*HR *Hazard ratio, *CI* Confidence interval, *DOAC* Direct oral anticoagulant, *ASA score* American Society of Anesthesiologists score

The variance inflation factor of the included covariates ranged between 1.045 and 1.102 and tolerance between 0.907 and 0.957, indicating low level of multicollinearity. The Cox proportional hazards model was statistically significant overall (*p* < 0.001, likelihood ratio test).

### Time to surgery

The time to surgery was significantly longer in the DOAC group compared to the non-DOAC group (median: 24.6 h [IQR: 19.4–34.7] vs. 22.6 h [IQR: 15.5–31.4], *p* = 0.0048) (Table 3). In only 5% of patients on DOAC the surgery started within 12 h, compared to 16% of patients not on DOAC (*p* < 0.001). Similarly, a significantly smaller proportion of the patients in the DOAC group had a time to surgery < 24 h (47% in the DOAC group vs. 56% in the non-DOAC group, *p* = 0.045). Contrary, no difference was observed for time to surgery within 36 h (76% vs. 79%, *p* = 0.4186) or 48 h (92% in both groups, *p* = 0.9668).

**Table 3:** Time to surgery in each study group. Time to surgery was defined as the time from the first diagnostic imaging confirming the diagnosis to surgery

	DOACs	No DOACs	*p* value^1^
Time to surgery (h), median (IQR)	24.6 (19.4–34.7)	22.6 (15.5–31.4)	0.0048
Surgery before 12 h, ***n*** (%)	8 (5%)	69 (16%)	< 0.001
Surgery before 24 h, ***n*** (%)	80 (47%)	238 (56%)	0.045
Surgery before 36 h, ***n*** (%)	130 (76%)	337 (79%)	0.4186
Surgery before 48 h, ***n*** (%)	157 (92%)	392 (92%)	0.9668

*DOACs* Direct oral anticoagulants, *IQR* Interquartile range

^1^p values were calculated using the Mann Whitney U test (for continuous variables) and the Chi-squared test (for categorical variables)

#### Transfusion requirements

The transfusion rates were significantly higher in the DOAC group of patients compared to those not on DOAC (51.2% vs. 39.4%, *p* = 0.009) (Table 4). However, there was no significant difference in transfusion volumes between the groups (median: 558 mL [IQR: 293–871] vs. 555 mL [IQR: 281.5–794], *p* = 0.091).

**Table 4:** Transfusion requirements in the DOAC and no DOAC group of patients

	DOACs	No DOACs	*p* value^1^
**Transfusion rates**, ***n*** **(%)**	87 (51.2%)	167 (39.4%)	0.009
**Transfusion volume (mL)**, **median (IQR)**	558 (293–871)	555 (281.5–794)	0.091

*DOACs* Direct oral anticoagulants, *IQR* Interquartile range

^1^ p values were calculated using the Mann Whitney U test (for continuous variables) and the Chi-squared test (for categorical variables)

## Discussion

### Mortality 360 days after surgery

The present study found a significantly higher mortality rate at 360 days in acute hip fracture patients treated with DOACs compared to those not treated with DOACs. This result is not unsurprising, given the significant differences in baseline characteristics between the two groups: DOAC-treated patients were older, had a higher proportion of males, higher BMI and comorbidity and frailty (as indicated by higher ASA scores and CFS, as well as NT-proBNP, creatinine and eGFR levels). These factors are well-established predictors of mortality and highlight the risk of confounding. To address this, a multivariate Cox proportional hazards regression analysis was performed. After adjusting for covariates, the negative effect of DOAC on mortality at 360 days remained, suggesting an independent association. However, residual confounding cannot be excluded.

Earlier studies show conflicting results regarding increased mortality in DOAC treated acute hip fracture patients [22–24, 28–30]. This is not entirely surprising since mortality rates may depend on local perioperative management practices and specific study populations, including variations in age, gender distribution, and comorbidities.

Several previous studies have found no significant mortality differences in AHF patients treated with DOAC and those who were not [28–30]. In contrast, other studies have reported a significantly higher one-year mortality among DOAC patients compared to non-anticoagulated controls [22, 23]. Contrary to the present study, these studies could not identify DOAC treatment per se as an independent predictor of mortality after adjusting for key covariates.

Further, a large Norwegian study reported both increased one-month and one-year mortality after adjusting for gender, age and Charlson Comorbidity Index in AHF patients on DOACs [24]. However, when further adjusting for time to surgery, the association between DOAC treatment and mortality disappeared. The authors suggested that these findings mean that the negative impact of DOAC use on mortality may be partially explained by its influence on prolonging the time to surgery.

In our study, mortality rate at 360 days was 35.9% in DOAC-treated patients, which is relatively high and comparable to previously reported one-year mortality [22, 23]. However, our findings are similar to the 360-day mortality found by Sundet et al. [24]. Although, as previously mentioned, the differences in study samples as well as treatment protocols make direct comparisons of mortality rates difficult.

### Time to surgery

In the current study, a significantly smaller proportion of the patients in the DOAC group had a time to surgery of less than 12 as well as less than 24 h. Additionally, patients using DOACs had a statistically significantly longer time to surgery compared to non-DOAC users. However, the median difference was only two hours, which does not seem to be significant from the clinical point of view. Since the reasons for surgical delay were not specifically investigated in this study, it remains unclear if the observed difference in time to surgery can be attributed solely to DOAC treatment. Our findings align with previous literature, which generally reports a delay in time to surgery for patients using DOACs compared to those without anticoagulants [31–33]. Notably, the extent of the difference varies greatly between these studies, with a median time to surgery for DOAC patients ranging between 27.6 and 66.9 h.

In contrast to these findings, some studies have reported no significant difference in time to surgery between DOAC users and non-DOAC users [29]. The varying results regarding time to surgery may reflect differences in sample characteristics, local protocols and perioperative management practices.

### Transfusion requirements

This study identified significantly higher transfusion rates in the DOAC-treated group compared to those not on DOACs, which is in concert with other studies [34]. Importantly, the significantly lower preoperative hemoglobin values among DOAC users, as well as differences in surgical methods, may both partially explain the higher transfusion rates observed in this group. Also, impaired hemostasis following trauma in anticoagulated patients likely contributes to greater blood loss and an increased requirement for transfusion.

Although the DOAC group patients had higher transfusion rates, it remains unclear if DOAC therapy is an independent risk factor for transfusion need. Earlier studies on geriatric hip fracture patients have found that preoperative anemia, gender, ASA score, hip fracture type, and BMI are independent risk factors for requiring blood transfusion [35]. The two groups in our study demonstrated significant differences in several of these factors, which highlights the potential for confounding and thus the need for cautious interpretation of these results.

Interestingly, a Danish nationwide cohort study found a slightly increased risk of transfusion among DOAC users compared to non-users even after adjusting for several covariates, including age, gender, BMI, Charlson comorbidity index, fracture type and surgical delay [28]. However, in this study the comparison was made with all non-users of DOACs, including those on antiplatelet or VKA therapy.

In contrast to these findings, other studies have not reported significantly higher transfusion rates in patients using DOACs [29, 36]. A study with 1714 patients did not find any significant differences in transfusion rates between DOAC and non-anticoagulated patients [23]. Furthermore, in this study a multivariate logistic regression analysis was performed, which did not find DOACs to be a significant determinant of blood transfusion.

The current study found no significant difference in transfusion volumes between the two groups when analyzing only patients who received transfusions. This suggests that while DOAC treatment may increase the risk of requiring transfusion, the volume per transfusion was similar between the groups. To the best of our knowledge, no prior studies have directly compared transfusion volumes between hip fractures patients on DOACs and those not on DOACs.

### Prevalence of DOAC use

The prevalence of DOAC use in our study population was 28.6%. Direct comparisons with earlier studies are difficult due to the varying inclusion and exclusion criteria. Additionally, the DOAC usage is rising which complicates comparisons with older studies [15].

A large Danish study conducted between 2015 and 2022 reported a DOAC usage of 16%, and a Norwegian study conducted from December 2016 to December 2017 reported a DOAC usage of 15% [24, 29]. Although both studies had similar inclusion and exclusion criteria to ours, they included patients on antiplatelet therapy in their non-DOAC group. This difference, combined with the fact that these studies were conducted a few years earlier, likely explains the slightly lower prevalence compared to our findings.

### Strengths and limitations

The strengths of this study include its reliance on objective, well-defined, and clinically relevant outcomes. Additionally, the use of Cox proportional hazards regression allowed for a robust time-to-event analysis, while adjusting for multiple covariates, which provided a closer assessment of the association between DOAC use and 360-day mortality. However, residual confounding cannot be entirely excluded, which is an inherent limitation of observational studies.

The retrospective study design may introduce information bias, since existing EHR were not designed for research. Consequently, the variability in the consistency, accuracy and completeness of data may affect the reliability of the results. Additionally, the retrospective design allows for reporting associations, but causality cannot be proven. Thus, there is a risk of unmeasured confounding factors.

Furthermore, the single-center setting of this study may limit its external validity. While the findings may not fully generalize to other populations with differing characteristics, the results should generalize reasonably for elderly AHF patients in Europe.

A further consideration is that differences in pharmacokinetics between DOAC types, such as half-life and elimination routes, could theoretically influence clinical outcomes. However, our sample size, as in most studies, did not allow for stratified analysis by DOAC type. It is possible that a sub-group analysis of DOACs with longer half-lives than apixaban could have shown clinically significant delays to surgery.

The Cox proportional hazards analysis lacks validation, thus there is a risk of overfitting. Although we attempted to address this issue by limiting the number of independent variables relative to the number of events (deaths) [26], the findings should be interpreted with caution. Furthermore, 74 patients were excluded from the Cox regression analysis due to missing data, which may introduce bias if the missingness was not completely random.

## Conclusions

The 360-day mortality, adjusted for covariates (age, gender, ASA score and CFS), was higher in AHF patients treated with DOACs vs. those without DOACs. This presumably reflects both perioperative challenges and the burden of underlying cardiovascular disease, which represents an inherent limitation when interpreting long-term outcomes. Also, this finding is likely influenced by residual confounding, which cannot be excluded. The time to surgery was longer and transfusion rates were higher in those treated with DOAC. These findings highlight the challenges of managing hip fracture patients on DOAC therapy and should inspire future research with a larger study population and multi-center setting. With further research a framework for individualized perioperative management protocols could be presented, which may improve outcomes for elderly acute hip fracture patients using DOACs.

## Appendix

**Table 5:** Electronic health records search criteria used to identify acute hip fracture patients, and all variables collected in the data collection process

EHR search criteria	Describing diagnosis: S72.0-S72.2 (ICD-10) Describing surgery: NFJ49-99, NFB09-99 (KVÅ, Classification of Health Care Measures)
**Variables collected**	Gender, age, height, weight, BMI, ASA score, CFS, NHFS, ICD-10 code, KVÅ code, time and length of surgery (min), type of anesthesia and surgery, time of admission and discharge, time to surgery (h), length of hospital stay (days), preoperative anticoagulation or anti-platelet therapy, polypharmacy (defined as 5 or more medications), intraoperative bleeding volume (ml), transfusion volume (ml), preoperative lab values (hemoglobin, creatinine, eGFR, prothrombin time, activated partial thromboplastin time, thrombocytes, NT-proBNP, DOAC concentration), lowest postoperative hemoglobin value, co-morbidity, and date of death

*EHR* Electronic health records, *ICD-10* International Statistical Classification of Diseases and Related Health Problems 10th Revision, *KVÅ* Classification of Health Care Measures, BMI Body mass index, *ASA score* American Society of Anesthesiologists, *CFS* Clinical Frailty Scale, *NHFS* Nottingham hip fracture score, *eGFR* Estimated glomerular filtration rate, *DOAC* Direct oral anticoagulant

## Data Availability

No datasets were generated or analysed during the current study.
